# Toilet revolution in China

**DOI:** 10.1016/j.jenvman.2017.09.043

**Published:** 2018-06-15

**Authors:** Shikun Cheng, Zifu Li, Sayed Mohammad Nazim Uddin, Heinz-Peter Mang, Xiaoqin Zhou, Jian Zhang, Lei Zheng, Lingling Zhang

**Affiliations:** a School of Energy and Environmental Engineering, Beijing Key Laboratory of Resource-oriented Treatment of Industrial Pollutants, University of Science and Technology Beijing, Beijing, 100083, PR China; b Department of Geography, Faculty of Social Sciences, University of Victoria, PO Box 1700 STN CSC, Victoria, BC V8W 2Y2, Canada; c EnviroSystems Engineering & Technology Co., Ltd., Tiangong Plaza A501, Xueyuan Road 30, Haidian District, Beijing, 100083, PR China

**Keywords:** Toilet revolution, Sanitation, Challenge, Opportunities, China

## Abstract

The wide-spread prevalence of unimproved sanitation technologies has been a major cause of concern for the environment and public health, and China is no exception to this. Towards the sanitation issue, toilet revolution has become a buzzword in China recently. This paper elaborates the backgrounds, connotations, and actions of the toilet revolution in China. The toilet revolution aims to create sanitation infrastructure and public services that work for everyone and that turn waste into value. Opportunities for implementing the toilet revolution include: fulfilling Millennium Development Goals and new Sustainable Development Goals; government support at all levels for popularizing sanitary toilet; environmental protection to alleviate wastewater pollution; resource recovery from human waste and disease prevention for health and wellbeing improvement. Meanwhile, the challenges faced are: insufficient funding and policy support, regional imbalance and lagging approval processes, weak sanitary awareness and low acceptance of new toilets, lack of R&D and service system. The toilet revolution requires a concerted effort from many governmental departments. It needs to address not only technology implementation, but also social acceptance, economic affordability, maintenance issues and, increasingly, gender considerations. Aligned with the ecological sanitation principles, it calls for understanding issues across the entire sanitation service chain. Public-private partnership is also recommended to absorb private capital to make up the lack of funds, as well as arouse the enthusiasm of the public.

## Highlights

• The history and current Chinese sanitation scenarios are presented.
• The concept of toilet revolution and the concrete toilet actions are elaborated.
• Challenges and opportunities for toilet revolution are discussed and analyzed.
• China provides valuable lessons-learned for the developing world.

## 1 Introduction – what's behind?

In 2015, one in three people (2.4 billion) in the world still used unimproved sanitation facilities, including 946 million people who still practised open defecation. Even in urban areas, where household and communal toilets are more prevalent, over 2 billion people use toilets connected to septic tanks that are not safely emptied or use other systems that discharge raw sewage into open drains or surface waters. Today over 880 million people are estimated to be living in slum-like conditions in the developing world's cities. About 50% of people living in rural areas lack improved sanitation facilities, compared to only 18% of people in urban areas. Poor sanitation around the world results in increased prevalence of diseases and pollution of the environment (MFA and UN, 2015, UNICEF and WHO, 2015). The World Bank estimates that poor sanitation costs the world 260 billion USD annually (Hutton, 2012). Poor sanitation contributes to 1.5 million child deaths annually from diarrhoea (Prüss-Ustün et al., 2014), which is the second leading cause of morbidity and mortality among children under the age of five, and the leading cause of death in sub-Saharan Africa (Lim et al., 2012, Murray et al., 2015, Walker et al., 2013). Excreta, grey water and solid wastes are the major contributors to the pollution load into the environment and pose a risk to public health (Katukiza et al., 2012). Public agencies often grapple with the question why the adoption of improved sanitation technologies has been slow (Seleman and Bhat, 2016).

**Fig. 1:**
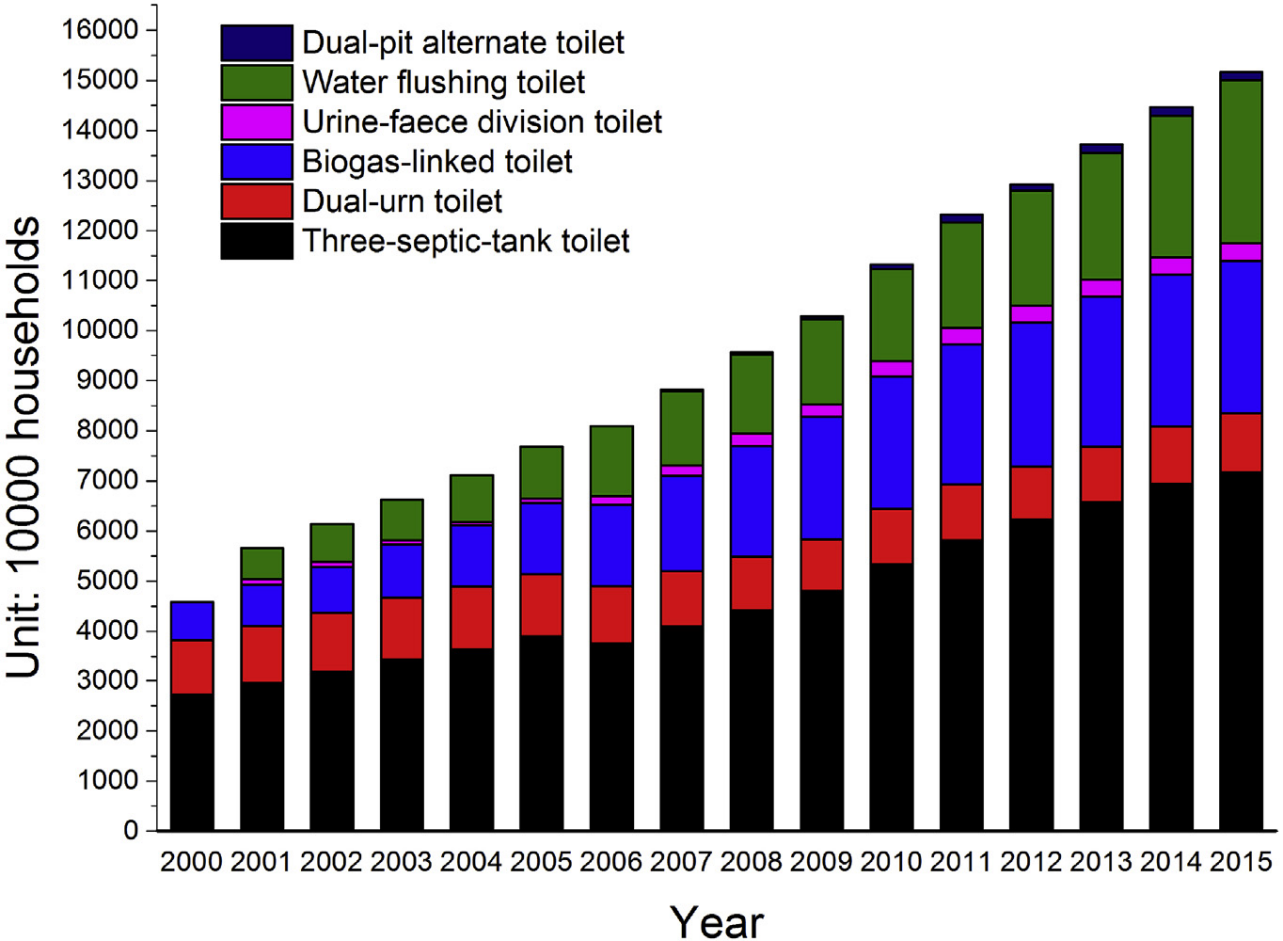
Yearly number of six different harmless sanitary toilets installed in Chinese rural areas from 2000 to 2015.

When it comes to China, the outlook is not optimistic either, although China had made great progress during the on-going toilet retrofitting action in rural areas. According to up-to-date official data (NHFPC, 2016), the coverage of sanitary toilets in rural areas has increased from 7.5% in 1993 to 78.5% in 2015, while the coverage of harmless sanitary toilets reached 57.5% by end of 2015. Fig. 1 presents the yearly number of six different harmless sanitary toilets, targeted by the government and installed in Chinese rural areas from 2000 to 2015 (NHFPC, 2015, NHFPC, 2016). However, 57 million households do not have their own sanitary toilet, but 40 million of those households among can use a public sanitary toilet. There are 17 million remaining households that still have serious hygiene issues resulting from poor toilets.

**Fig. 2:**
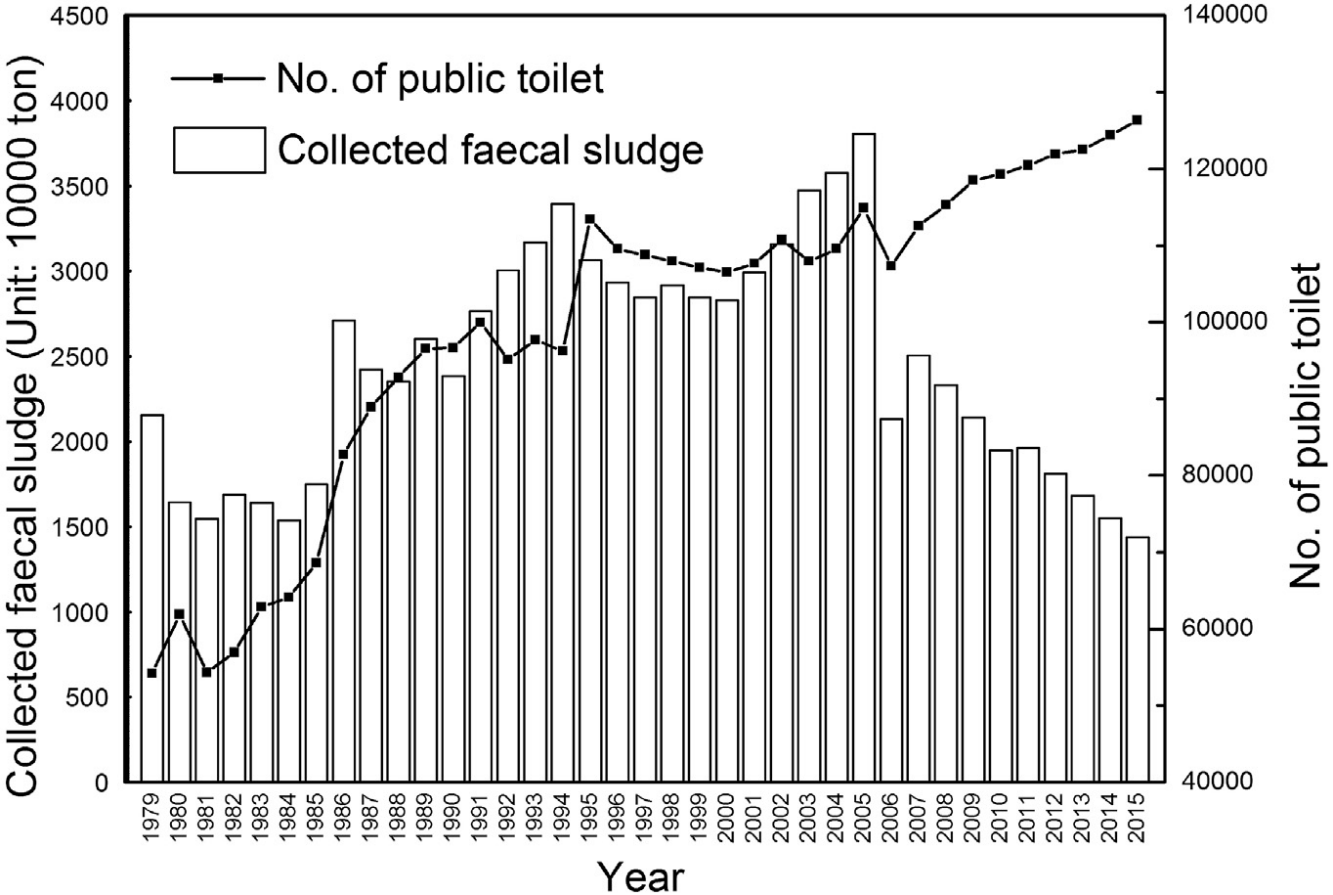
Collected faecal sludge and quantity of public toilet in urban areas.

In light of urban sanitation, in 2015, the collected amount of urban faecal sludge was 14.28 million tons. Among this, 6.76 million tons was treated, while the treatment ratio was 47.3%. Of all the provinces and municipalities, Beijing led the nation in that the treatment ratio of faecal sludge reached 92.3%. Fig. 2 shows the trend of collected faecal sludge and quantity of public toilets in urban China (MOHURD, 2016). It is observed that the amount of faecal sludge has decreased in the past decade. The reason may come down to the greater distribution of municipal pipelines which can collect more human faeces into wastewater treatment plants. Another reason would be that government contracts with private companies for collection and handling of faecal sludge are not under governmental responsibility and so are not taken into statistics. However, this doesn't mean that the actual faecal sludge amount and its potential damage to the environment is reducing.

## 2 What is going on? – toilet action

When foreigners visit China tourist areas, they complain about the issue of public toilets the most. Many foreigners said they will never forget the scary toilet experience. Given this fact, how could our tourism industry take big strides?(China Daily, 2015) Thus, the tourism sector has fired the first shots in the toilet revolution. The China National Tourism Administration (CNTA) set the target that from 2015 to 2017, 25,000 public toilets will be upgraded and another 33,500 will be newly built in tourist areas within 3 years. This is also known as the Three-year Toilet Plan (CNTA, 2015). Clean and standard toilets will be a key index for evaluating tourism areas. It was reported that 89.33% of the task had been finished by Feb of 2017 (CPRI and CGPI, 2017).

“Toilet Revolution” became a hot word in 2015 in China. On 1st of April 2015, President Xi Jinping made important comments on toilet revolution and civilized tourism. In addition, when he visited in Jilin Province on 16th of July 2016, he saw that some farmers still used traditional latrine pits. He said China's rural areas would also launch a “toilet revolution” to let farmers use sanitary toilets. When talking about toilets in rural China, there would be two barriers, one is bad odour, the other is hidden sanitary trouble. Actually, the toilet revolution is tightly associated with the patriotic health campaign, which first started in the 1950s and aimed to improve sanitation and hygiene, as well as attack diseases (Yang, 2004). Since 2004, the central government has earmarked RMB 8.64 billion which has renovated 21.03 million rural toilets. The scenarios of six different harmless sanitary toilets are depicted in Table 1. The goal for rural toilet retrofitting in China is to reach the 85% popularizing rate of sanitary toilets by 2020 (NPHCC, 2015)and 100% by 2030 (State Council, 2016). Chronology of toilet plans and actions is presented in Table 2.

**Table 1:** Scenarios of six different harmless sanitary toilets (Hu et al., 2016).

Type of toilet	Characteristic	Sketch	Suitable area
Three-septic-tank type	It is composed of three septic tanks. The wastewater flows one by one. The first chamber is designed as settler, the second as post-settler and scum separator, and the third as storage tank, size of last tank depends seasonal reuse pattern or other available emptying services.		Most of rural areas, mainly South of China. If in North of China, freezing protection should be respected. In some areas, the tanks are emptied by vacuum trucks and the content is centralized treated.
Double-vault funnel type	The vault is prefabricated by ceramic, cements or composite. The installation is easy. Needs post-composting of liquid before used as fertilizer or only used as soil improve before planting seasons.		Huai River basin, middle and lower streams of Yangtze River, North China Plain, northwest and southwest arid areas where is short of rainfall.
Double pit alternate type	The two pits work alternately. When one is used, the other is stored for composting by correcting the C/N ratio.		Northwest and southwest arid areas where is short of rainfall.
Biogas-linked toilet	The toilet is built next to livestock shed. Human waste and animal waste are both used for biogas production, which is used mainly for cooking and lighting.		Nationwide, especially south and west of China where climate is warm.
Urine-faeces division toilet	Urine and faeces are collected separated, urine can be diluted with water and used for fertilizer directly. Faeces are dehydrated for harmless treatment.		Nationwide, especially arid and water-deficient area
Integrated flushing toilet	The toilet is connected with fresh water supply pipe and complete drainage connect for sewage treatment. It requires building sewage pipeline and treatment facility, so the overall cost is high.		Nationwide, rich rural areas or suburbs which are connected to sewer pipeline network combined with central or decentralized treatment station.

**Table 2:** Chronology of toilet plans and actions in China.

Year	Action/Plan/Regulation	Leading department	Remark
1950s	Start of National Patriotic Health Campaign	State Council	Establish Patriotic Health Campaign Committee at all levels
1970s	Two Management and Five Retrofitting Action	National Patriotic Health Campaign Committee (NPHCC)	Management of human manure and water supply, retrofitting of water well, toilet, animal pen, stove and living environment in rural areas
1980s	Three-in-One Patriotic Health Campaign	NPHCC	Combine water supply, toilet retrofitting, and health education into one whole.
1992	Outline of the Program for Chinese Children Development in 1990s	State Council	List rural toilet retrofitting into the program
1997	Decision on Sanitation Reform and Development	State Council, Central Committee of the Communist Party of China	List rural toilet retrofitting into the work plan
2000	Ecological Household Enrichment Plan	Ministry of Agriculture (MOA)	Connect toilet with biogas digester, known as biogas-linked toilet
2001	Outline of the Program for Chinese Women Development 2001–2010	State Council	List rural toilet retrofitting into the program
2002	Decision on Further Accelerating the Rural Sanitation Work	State Council, Central Committee of the Communist Party of China	Focus on retrofitting toilet and water supply in rural areas to mobilize renovation of rural environment, in order to prevent disease.
2009	Opinions on Deepening Medical and Health System Reform	State Council, Central Committee of the Communist Party of China	List rural toilet retrofitting into national major public health service program.
2009	Key Implementation Plans on the Reforms of the Medical and Health Care System in Recent Period (2009–2011)	State Council	List rural toilet retrofitting into national major public health service program.
2010	National Urban and Rural Environmental Sanitation Clean Action Plan (2010–2012)	NPHCC	Popularize rural sanitary toilet to make coverage increase 10% within 3 years.
2011	Outline of the Program for Chinese Women Development 2011–2020	State Council	List rural toilet retrofitting into the program, set the target that coverage of rural sanitary toilet will reach 85% by 2020.
2015	National Urban and Rural Environmental Sanitation Clean Action Plan (2015–2020) (NPHCC, 2015)	NPHCC	Set the target that coverage of rural sanitary toilet will reach 85% by 2020.
2016	Health China 2030 Program Planning (State Council, 2016)	State Council, Central Committee of the Communist Party of China	Set the ambitious target that coverage of rural sanitary toilet will reach 100% by 2030.

Internationally, in 2011, the Bill & Melinda Gates Foundation (BMGF) initiated the Reinvent The Toilet Challenge (RTTC) to bring sustainable sanitation solutions to the 2.4 billion people worldwide who do not have access to safe, affordable sanitation. Grants have been awarded to sixteen researchers around the world who are using innovative approaches—based on fundamental engineering processes—for the safe and sustainable management of human waste. In addition to these RTTC grants, BMGF has made a range of other investments that are aligned with reinventing the toilet, and we are continuously seeking to expand our partnerships on this challenge. In August 2013, the foundation announced the Reinvent The Toilet Challenge – China (BMGF, 2013). The foundation would invest US$5 million to support Chinese investigators to drive research, development, and production of the “next generation toilet” (BMGF, 2013). This China toilet challenge is an effort targeted to a specific country after India and is a testament to the research and development capabilities in China. After two-round selections, nine proposals have been funded finally (RTTC-China, 2017). Domestically, CNTA, BMGF and University of Science and Technology Beijing (USTB) launched the toilet innovation programs in recent years (Table 3), in response to the “Toilet Revolution” and speed up the progress.

**Table 3:** Toilet innovation actions in recent years.

Year	Activity	Scope	Initiator
2011	Reinvent the Toilet Challenge (RTTC)	Global	BMGF
2013	Reinvent the Toilet Challenge-China (RTTC-China)	Chinese enterprise/academy	BMGF
2015	1st National Tourism Toilet Design Competition	Chinese enterprise, academy, private	CNTA
2015	1st Reinvent Toilet Contest for Chinese Students	Chinese college	BMGF, USTB
2016	1st National Toilet Technical Innovation Competition	Chinese enterprise/academy	BMGF, CNTA

## 3 What's toilet revolution?

Whether you call it the loo, john, privy, lavatory or toilet, this facility is essential wherever humans gather or live: toilet provision has even been called the barometer of civilization (Stanwell-Smith, 2010). However, toilets have always been treated as a matter of taboo, especially any form of latrines, while flush toilets are considered prestigious and desirable. There is no explicit literature report on the origin of the toilet revolution in China. Actually, the parlance “toilet revolution” was proposed first by UNICEF in 1997, when UNICEF and NPHCC cooperated to promote toilet retrofitting in China. It referred in particular to toilet retrofitting in developing countries.

Currently, the concept of a toilet revolution is enlarged and extended. It happens in many sectors, for instance, toilet retrofitting in rural areas, public toilets in tourist areas, public toilets in highway resting areas, reinvented toilets in R & D, etc. Moreover, it is not confined to the toilet itself, but to the whole sanitary system. A sanitation system – compared to a sanitation technology – considers all components required for the adequate management of human wastes, such as storage, collection, transport, treatment, discharge or reuse at the following levels (Zurbrügg and Tilley, 2009). Starting at the household level with waste generation, a system can include storage and potentially also treatment and reuse of all products such as urine, excreta, greywater, rainwater/stormwater or even solid waste (Schmitt et al., 2017). However, problems can rarely be solved at the household level alone. The household “exports” waste to the neighbourhood, town, or downstream population. In such cases, it is crucial that the sanitation system boundary is extended to include these larger spatial sections.

Looking back, the concept of the toilet revolution is somehow equal to the concept of ecological sanitation (Eco-San) (Hu et al., 2016, Winblad and Simpson-Hébert, 2004). The term ‘Eco-San’ appeared in the 1990's (Esrey et al., 1998), and quickly got a shot at stardom with the new millennium concepts. The UN issued a declaration of ‘Eco-San - closing the loop in wastewater management and sanitation’ in 2000 (Winblad, 2004). The Eco-San system is an alternative approach to realize sustainable sanitation, which is closely associated with toilets because toilet-based source separation always facilitates greater resource recovery as an alternative solution. Source separation of wastewater flows, such as blackwater (i.e. wastewater from toilets) and urine, captures concentrated nutrient-rich waste which makes nutrient recovery and pollutant removal more efficient (Larsen et al., 2013, McConville et al., 2017, Simha et al., 2017). Eco-San is known as the resource-oriented sanitation and is based on ecosystem approaches, the closure of material flow cycles, a novel trend of pollution treatment (from sewage disposal to resources reclamation), and a re-conceptualization of sanitation (from a ‘drop-flush-forget’ mode to environmental protection at sources by means of ‘drop and reuse’ mode) (Haq and Cambridge, 2012, Langergraber and Muellegger, 2005). It can be seen that the toilet revolution, from the viewpoint of Eco-San, is dedicated in optimizing cost efficiency, resource recovery and waste disposal (Werner et al., 2009).

To sum up, toilet revolution comes down to the definition that “toilet revolution is a step-wise campaign which tries to ensure hygienic separation of human excreta from human contact, to provide sanitary and comfort space for users, to prevent human excreta from pollution of environment, and to realize the resource recycling.”

## 4 Opportunities for implementing the toilet revolution

China owns the biggest toilet market in the world. Some factors can promote a toilet revolution, increasing the likelihood.

1. Millennium Development Goals and Sustainable Development Goals

At the beginning of the new millennium, the United Nations (UN) Millennium Development Goals (MDGs) unveiled a special horizon - one that the entire developing world has been tasked to arrive at by 2015. However, the world must first cross the water barrier to fulfil the task (ADB et al., 2006). On 25th September 2015, world leaders gathered at the UN in New York to adopt the 2030 Agenda for Sustainable Development, which comprises 17 new Sustainable Development Goals (SDGs). The new SDGs, and the broader sustainability agenda, go much further than the MDGs. Among them, Goal 6 ensures availability and sustainable management of water and sanitation for all (UNDP, 2015). By 2030, it aims to achieve access to adequate and equitable sanitation and hygiene for all. It also aims to end open defecation, paying special attention to the needs of women and girls and those in vulnerable situations. In addition, it requires to improve water quality by reducing pollution, eliminating dumping and minimizing release of hazardous chemicals and materials, halving the proportion of untreated wastewater and substantially increasing recycling and safe reuse globally (UNESCAP et al., 2015).

Globally, at least 1.8 billion people use a source of drinking water that is fecally contaminated (UNDP, 2015). Providing reliable and affordable sanitation facilities in rural areas is a challenge in many parts of the world, particularly in developing countries. As roughly estimated, there is approximately 9 billion tons of domestic wastewater discharged every year in rural areas of China (Zhou et al., 2008). The world is striving to meet MDGs and SDGs and China is no exception. Popularization of improved sanitation facilities can undoubtedly increase the possibility of achieving some of the MDGs and SDGs. i.e. Target 3 (by 2030, end the epidemics of AIDS, tuberculosis, malaria, and neglected tropical diseases and combat hepatitis, water-borne diseases, and other communicable diseases), and Target 6 (by 2030, achieve access to adequate and equitable sanitation and hygiene for all and end open defecation) (UN, 2016).

In such cases, a toilet revolution can help to ensure environmental sustainability and reverse the loss of environmental resources. It does not imply overexploitation of the existing resources, but improving their management by reducing, recycling and reusing human wastes (Libralato et al., 2012).

2. Government support at all levels

Since the 18th National Congress of the Communist Party of China, the central government indicated the future development direction for the whole nation that infrastructure construction and social undertaking will give priority to rural areas. Sanitation is important to implement the Socialism New Countryside Construction. The popularization of sanitary toilets is helpful to improve rural living conditions and promote rural civilization, and thereby achieve the goal of building a moderately prosperous society.

Toilet revolution has aroused leaders' attention. President Xi Jinping has given special instructions on the toilet revolution and civilized tourism in 2015. Premier Li Keqiang has also called for innovation in the country's patriotic health campaign (The State Council, 2015). The campaign “plays an irreplaceable role” in preventing and controlling the spread of disease, improving hygiene in urban and rural areas and strengthening public awareness. Toilet revolution has been listed as the Number 1 prioritized work of CNTA since 2015.

Under central government, local governments built up “Leading Group for Toilet Revolution”, which is in charge of formulating and promoting a toilet and sanitation improvement plan. For instance, eight provinces have held deployment meetings for tourist toilets, eleven provinces have been building up province-level leading and coordinating groups for a tourist toilet revolution up to now. On the 2015 World Toilet Day, CNTA, Ministry of Housing and Urban-Rural Development (MOHURD) and the Beijing government, held the activity of “China Toilet Revolution Mobilization Day”. On 1st April 2016, CNTA and NPHCC together held the activity of “China Toilet Revolution Advancing Day”. These activities all indicate the strong support from government at all levels.

3. Environmental protection and resources recovery

Urban-industrial growth is beginning to skew China's water allocation balance. Already, competing demand for water is turning this resource into a basis for conflict (Narain, 2012). China suffers a lot from environmental pollutants in the form of wastewater. In 2015, the total amount of COD discharge reached 22.2 million tons, the total nitrogen reached 4.5 million tons while the total phosphorus reached 5.3 million tons. Even though pollutant discharges had declined year by year since 2012, the total amount of pollutants is still huge (MEP, 2016).

In terms of the agriculture sector, the agricultural pollution sources exceeded industrial pollution sources in 2015 for the first time. MOA set the target that the use of chemical fertilizers and pesticides should become zero-growth by 2020. That means, in the coming few years, the use of chemical fertilizers will still increase until 2020. The up-to-date statistics show that the total amount of chemical fertilizer applications in agriculture reach 60.0 million tons, among which, nitrogenous fertilizer reaches 23.9 million tons (in N), phosphatic fertilizer reaches 8.45 million tons (in P_2_O_5_), potassic fertilizer reaches 6.42 million tons (in K_2_O), the remainder is compound fertilizer (MOA, 2015).

Annually, an individual can produce as much as 5.7 kg of N, 0.6 kg of P and 1.2 kg K which are key ingredients found in chemical fertilizers (Esrey et al., 1998, Karak and Bhattacharyya, 2011). More than 90% of nitrogen and phosphorus come from human excrement in the form of urine and faeces (Fittschen and Hahn, 1998, Larsen and Gujer, 1996). Compared with the amount of chemical fertilizer applications, if the valuable nutrient elements are collected and recovered in agriculture, this can replace 20% of chemical fertilizer by rough calculation. The community associated great benefits from using human excreta in agriculture, especially if composted, and did not associate risks with the use of composted excreta if it was dry and lacked odour (Mackie Jensen et al., 2008). Empirical research has shown that the use of manure significantly improves crop yield, soil fertility and water and moisture conservation (Andersson et al., 2016, Liu et al., 2014).

4. Diseases prevention and poverty alleviation

Modern medicine indicates that human faeces contain many kinds of pathogens which can cause serious intestinal infectious diseases and parasitic diseases. The amount of untreated faeces sludge discharged into the open environment poses a serious public health risk. For instance, the WHO reported that poor sanitation contributes to 1.5 million child deaths from diarrhoea each year. Chronic diarrhoea can also hinder child development by impeding the absorption of essential nutrients that are critical to the development of the mind, body, and immune system. It can also impede the absorption of life-saving vaccines (Bassan et al., 2014).

The potential for pathogen contamination are high, since faeces is the greatest source within the components that make up conventional wastewater (Skambraks et al., 2017, Vinnerås et al., 2006). A 5 m^3^ truck load of faeces sludge dumped into the environment is the equivalent of 5000 people practicing open defecation. The consequences of this waste from open defecation entering the environment are staggering. In addition, pathogens have been known to be a major constraint when using wastewater products in agriculture, and since faecal sludge can be highly contaminated, this is a key factor for implementing sanitation systems, which aim to reuse these wastewaters (Afolabi and Sohail, 2017, Magri et al., 2015).

The Toilet Revolution aims to achieve popularization of sanitary toilets, which would play an important role in disease prevention. For instance, biogas sanitary toilets can kill a considerable percentage of pathogens inside human excreta (Wu and Xu, 2003). A study was carried out by Sichuan Province Institute of Parasitic Disease Prevention and Control to test the treatment effect of biogas-linked sanitary toilets in six projects. Generally, Faecal coliforms of treated sewage was >10^−4^. The number of parasitic ovum ranged from 0.565/L–1.074/L. BOD <50 mg/L. SS < 60 mg/L. Chromaticity was <100. These indicators could meet the requirement of the Integrated Wastewater Discharge Standard (GB8978-2002) and Sanitary Standard for the Non-hazardous Treatment of Night Soil (GB7959-1987) (Zheng et al., 2006).

As estimated, the input-output ratio is approximately 1:5.3 for retrofitting a sanitary toilet (NPHCC, 2014). The benefit mainly attributes to the diseases prevented and health improvements made (Mills and Cumming, 2016). The World Health Organization estimates that poor sanitation costs the world 260 billion USD annually (Hutton, 2012). Due to diseases prevention, cost on health-care and medicine will be reduced, thus, implementing a toilet revolution will contribute to alleviate poverty and improve wellbeing.

## 5 Challenges for implementing toilet revolution

Many challenges still exist and must be overcome for sound development.

1. Insufficient fund and policy support

The independent support policy on a toilet revolution is still missing, although some subsidy policies have been introduced at local economic development level. The absence of incentive policies makes social financing difficult. Enterprises and research institutes should be encouraged to be involved in a toilet revolution by incentive policies, such as tax preferences. In terms of local government initiatives, it is suggested that the toilet revolution is integrated into the assessment index system of a government's achievement. This can vastly mobilize the initiative of local governments and urge them to formulate related regulations and plans to implement a toilet revolution. What's more, toilet revolution can be integrated into social and economic development plans.

All kinds of toilets require initial funds, let alone the following treatment system. By rough estimation, the toilet revolution requires billions of CNY for new construction. In some cases, when a toilet is damaged by natural disasters, the repair of the toilet is also short of funds. The fund shortage has become a bottleneck for promoting rural sanitation systems, especially in less-developed areas. The main fund support is from national subsidies, the market is still at the rudimentary stage (Gao et al., 2014). For instance, the sanitary toilet retrofitting can only be subsidized by the government. What is worse is that there is no specific subsidy for the toilet itself, it has to normally be attached to a household biogas program, which is under a national debt program. The toilet revolution is often associated with Three Rural Issues (issues about agriculture, farmers and rural areas) or Socialism New Countryside Construction, public health, poverty alleviation, environment protection, etc. In such cases, the fund from other fields can be partly transferred towards toilet revolution.

2. Region imbalance and lagging approval process

There is an imbalance of development of sanitary facilities with considerable urban-rural and regional gaps. The penetration rate of sanitary toilets in central and western parts of China are obviously lower than those of eastern regions. Poor sanitation normally exists in poor areas, where the burden of inadequate sanitation is greatest. By 2020, the coverage of rural sanitary toilets should reach 85%, which can be treated as the national average target. When it comes to provinces, five developed municipalities/provinces including Beijing, Tianjin, Shanghai, Jiangsu, and Zhejiang will reach 100%, with another twelve provinces on track to reach 82%. There is no specific goal for Tibet currently (NPHCC, 2014). This can indicate the imbalanced development of sanitary facilities. In some villages, people want a sanitation technology that requires more water just to be able to bring pressure on the government for an improved water supply. In some villages, people reject water-intense sanitation technologies for lack of water (Liu et al., 2016).

Toilet revolution involves multiple departments. At the level of the State Council, the ministries include the National Health and Family Planning Commission (NHFPC, i.e. former Ministry of Healthy), CNTA, MOHURD, Ministry of Environmental Protection (MEP), MOA, Ministry of Transport (MOT), and Ministry of Land and Resources (MLR), etc. Generally, CNTA is in charge of toilets in tourist areas, which is reported most by news media. MOT is in charge of toilets at the rest areas of highways or railways. NHFPC and MOA are in charge of sanitary toilet retrofitting in rural areas, MOHURD is in charge of public toilets in cities. MEP is in charge of environmental impact assessments for most toilets. MLR is in charge of land use for toilet construction. However, when one department (e.g. CNTA) implements its own aspect, it also needs to coordinate with other departments. It must face a series of approval processes from each department. In such cases, overall coordination is important. The high-level coordination group from central government which can coordinate all these ministries seems to be missing. At this moment, central government establishes more than 20 leading groups in key issues. For instance: the comprehensively deepening reform leading group; the network security and informatization leading group; the addressing climate change and energy conservation and emissions reduction leading group; etc. It is also suggested to establish a toilet revolution leading group, which can coordinate the different departments. In addition, the “green channel” for the toilet project should be open, in order to shorten the construction time, especially, the approval process for land use.

3. Weak sanitary awareness and low acceptance of new toilets

The public think that environmental pollution has become a serious problem for China (Jingling et al., 2010). Public awareness towards the problem of wastewater pollution has grown tremendously in recent years (Wolters et al., 2016). However, the education gap between urban area and rural areas is still huge. Due to the limitation of education levels, toilet and human waste are always treated as taboos: people do not like talking about toilets in public. In addition, there are still many uncivilized phenomena regarding the use of toilets in public areas, e.g. tourist toilets. Some people do not take care of public facilities so much, they only focus on their own sanitation maintenance but neglect the public environment. The early education had a significant association with whether the study households had an improved toilet or not. It was evident that education, water supply and sanitation all had some connection with each other. Therefore, a strong collaboration between agencies that are in charge of elementary education, water supply, sanitation, and public health is necessary for implementing sanitation technology.

Another issue for improving sanitation involves acquiring a sound knowledge of the feasible sanitation systems and technologies which, in the site specific context, can achieve the intended objectives of health, hygiene and wellbeing improvements (Zurbrügg and Tilley, 2009). In spite of many benefits, the lack of knowledge and awareness of new toilets remains a barrier to their acceptance and implementation. Engineers and the water/construction industry are resistant to accepting a new toilet they are unfamiliar with (Cordova and Knuth, 2005). With the new sustainability paradigm of the 21st century, interest in new toilets has been growing. With this growing interest there are still gaps in knowledge about the engineering of sanitary toilets, it is now timely to revisit the status of sanitary toilets and bring awareness to this technology so they can be better evaluated for possible adoption as an alternative sustainable sanitation system. Conventional water flushing toilets are still the mainstream technology. In the past, sludge management from onsite facilities has not been a priority of engineers or municipalities, and has traditionally received little to no attention. Several generations of engineers have considered waterborne, sewer-based systems as the most viable, long-term solution to fulfil sanitation needs (Dodane et al., 2012). This lack of awareness and negative image of new toilets is likely to arise from insufficient experience and literature. There are only a few success stories regarding new toilets in low-income countries. The public may not accept the technology because of perceived odour and maintenance issues, which are the key factor for neglecting new toilets (Ramani et al., 2012).

4. Lack of R&D and service system

Diffusion of toilets as a pro-poor innovation is a challenge because their successful adoption calls for a change in individual behaviour, daily routines and perhaps even social norms (Ramani et al., 2012). Toilet technology is always the primary topic in the R&Ds of the toilet revolution. By far, the most well-known R&D activity is RTTC-China, launched by BMGF. Although it has been well received, the popularity is limited because the innovations BMGF supports can be most immediately valuable in densely populated areas, its main focus is on urban sanitation. This is far from enough for the Chinese toilet revolution. R&D on the toilet revolution contains process design, device development, ergonomic human engineering, psychology, behavioural science, etc. Many aspects are still at the starting stage. The support from the Ministry of Science and Technology is missing, and in such a case, China has not formed a sound R&D environment nationwide.

Once one sanitation system is built, a service chain makes access to sanitation a reality. For instance, without collection and transport companies to remove faecal sludge, onsite systems will not function properly. The matched service supporting system includes a technical consulting service, operational training service, resident publicity service, public and household facility maintenance service. One barrier to low persuasion of sanitary systems is the lack of successful projects, which can be overcome by better maintenance based on a well-established service system. Construction attributes less to sustainable operation of a sanitary system than maintenance. However, qualified construction is the prerequisite to subsequent convenient maintenance. Jerry-built toilet must be avoided in the beginning, so a strict acceptance inspection is strongly recommended. In addition, toilet maintenance could be outsourced by specialized team, which can be paid in accordance with its service. The users' feedback should capture attention as well.

## 6 What to do next?

The toilet revolution is a nationwide action. Institutionally, the toilet revolution should be integrated into social reform and new countryside construction, which is highlighted by central government. Toilet revolution requires concerted effort from many departments. It is recommended that one department or organization should be built solely aiming at implementing toilet revolution. It should be responsible for coordinating each department, managing local toilet and sanitation systems, including the decision-making, R & D, design, manufacture, and maintenance of sanitation systems: in a word, considering sanitation systems from cradle to grave. A new system should be built, which integrates urban and rural sanitation facilities. Meanwhile, a complete management and service network should be structured to maintain the high-efficiency and sustainability of the sanitation system. In terms of R&D, integration of orientalism and developed technology, modern technology and traditional custom, high-tech technology and common appropriate technology should be taken into consideration carefully.

Providing sustainable solutions for toilet revolution needs to address not only technology implementation but also cost, ownership and maintenance issues. Technologically, solving the sanitation challenge in China will require radically new innovations that are deployable on a large scale. Innovation is especially needed in densely populated areas, where billions of people are only capturing and storing their waste, with no sustainable way to handle it once their on-site storage—such as a septic tank or latrine pit—fills up. Ground-breaking improvements in toilet design, pit emptying, transportation method (Fan et al., 2017), and sludge treatment, as well as new ways to reuse waste, can help governments and their partners meet the enormous challenge of providing quality public sanitation services. There is much technical guidance available free of charge via the internet for designing and improving complete access to environmental sanitation. Considering the scenarios in China, especially aiming at toilet revolution, readable resources in Chinese are in shortage. Concept of toilet revolution should be propagandized step by step.

Toilet revolution should be well aligned with the Eco-San principles. The Eco-San aims to meet socio-economic requirements (Uddin et al., 2014), prevent pollution of surface and ground water, sanitize urine and faeces, recover nutrients for food production, and save water, energy and resources in a given local context. By being decentralized, requiring little to no water, and producing a value product (fertilizer), ecological toilets offer good promise as a sustainable solution to water and wastewater infrastructure issues (Lens et al., 2001, Mankad and Tapsuwan, 2011, Sasse, 1998). When applied to the water and sanitation infrastructure, ecological design principles point to human dimension (e.g. incorporating stakeholders in design), learning from nature (e.g. decentralization; elimination of the concept of waste; meeting multiple functions such as treating human waste while producing a value product, limited energy input to the system, and system design specific to location and scale), and integrating nature (e.g. relying on nature's processes for treatment) (Apul, 2010). It is considered more ready and suitable to be applied in rural areas, where the residence is more decentralized and nearer to farmlands than urban. Sustainability with respect to sanitation implies that the system needs to comprise of collection, storage, transport, and treatment of human excreta, grey water, solid waste and rainwater/stormwater, as well as the safe disposal or reuse of end products (Katukiza et al., 2012). A sustainable sanitation system should be technically feasible, acceptable to the users, affordable and contribute to health improvements and environmental protection. Population density, settlement patterns, landscape, water availability, household incomes, ownership and socio-cultural issues are also key factors that cannot be ignored. Sustainability of sanitation also requires institutional structures and arrangements to be in place for operation, maintenance and upscaling of interventions (Chinyama et al., 2012).

A toilet revolution needs to provide everybody with access to improved sanitation and sanitary toilets, irrespective of whether the area is rural or urban, the people rich or poor or the toilet private or public. In rural areas, the six kinds of harmless sanitary toilets should be promoted as before. It is encouraged to construct a four-chamber eco-toilet and biogas digester, and thereby strengthen the harmless use and resource-oriented use of human faeces. New-built housing and government-subsidized housing in rural areas should be attached to harmless sanitary toilets. Public sanitary toilets should be popularized at township government buildings, primary and middle schools, health clinics in towns and townships, rural community integrated service stations, pedlars' markets, tourist attractions, highway roadsides, etc. Health education should be highlighted. Farmers should be guided to use sanitary toilets, and the long-term effect management mechanism should be formed on the build-maintenance-use of sanitary toilets.

The toilet revolution has evolved from a purely technical discipline to one that includes social, environmental, economic and, increasingly, gender considerations (Tilley et al., 2013). The gender distribution for public toilets should be re-considered. It is inspiring that toilet design specification has been modified for future planning and design. Currently, the cost of new toilets (e.g. vacuum or air-flushing toilet) is estimated from the purchase of a new toilet, which is higher than a flush based toilet. From a user's perspective and in presence of low water and sewer utility rates, new toilets are not currently economical. Therefore, the cost is a barrier from a building designer or a home owner perspective. However, the true cost of large scale use of new toilets is not known since system level analyses comparing new toilets to centralized infrastructures have not been researched. So research on scaling up of demonstration projects should be conducted.

Public toilet services have traditionally been under public procurement provision. Unfortunately, there are many experiences in which public provision failed to achieve acceptable results. There are many experiences public-private partnerships (PPP) in this sector worldwide and PPP has become the buzzword in wastewater and solid waste management circles. PPPs are long-term contracts between the public and private sector in which the private sector has responsibility for significant aspects of the building and operation of an infrastructure for the delivery of public services that the public sector should provide while both sectors share risks, costs and benefits (Arbulú et al., 2016, Johannessen et al., 2014). Toilet revolution can also introduce PPPs and absorb private capital to make up the lack of funds, as well as arouse the enthusiasm of the public. Prevailing opinion is that inadequate toilet and sanitation infrastructure is not a problem. The problems are a lack of investment in creating infrastructure and a lack of managerial capacities to operate the systems, once created. The argument leads logically to defining the meaning of toilet revolution. On one hand, there are infrastructure projects created via private investment, through concession agreements and, on the other, there is handing over of public water systems to private parties to operate and “maximise efficiency” (Narain, 2012).

Finally, the sanitation control of human pathogens in effluent should gain more attention. This is because health should not be jeopardized by residual pathogens remaining in the water after treatment (WHO, 2016, Winward et al., 2008). Even though various wastewater treatment technologies including centralized and decentralized systems have been developed, the overall treatment capacity is still relatively low in developing countries due to the economic concerns (Wu et al., 2016). Appropriate technology should be highlighted and employed to fulfil the end requirements of the toilet revolution. For instance, composting toilets require little to no water and can therefore disconnect the toilet from both the water supply and wastewater infrastructure (Anand and Apul, 2014), a biogas toilet can make use of human waste and other household organic waste to produce biogas for cooking and return the digestate to farmland as fertilizer. Meanwhile, pathogens are killed in the digester which results in sanitary disposal of human waste (Mang and Li, 2010).

## 7 Conclusions

Improved sanitation—including waste treatment and resource recovery—is essential to a healthy and sustainable future for the developing world, with China as no exception. Toilet revolution in China is not just a buzzword, instead, it integrates environmental protection, disaster prevention, resource recovery, and sustainable development into a consolidated whole. Toilet revolution requires understanding issues across the entire sanitation service chain, including waste containment (toilets), emptying (of pits and septic tanks), transportation (to sewage treatment facilities), waste treatment, and disposal/reuse. MDGs and SDGs, government support, environmental protection and resource recovery provides opportunities for implementing the toilet revolution in China. Meantime, the challenges faced are: insufficient funding and policy support, regional imbalance and lagging approval processes, weak sanitary awareness and low acceptance of new toilets, lack of R&D and service system. The toilet revolution in China requires a concerted effort from many governmental departments. It needs to address not only technology implementation, but also social acceptance, economic affordability, maintenance issues and, increasingly, gender considerations.
